# Gemifloxacin, a Fluoroquinolone Antimicrobial Drug, Inhibits Migration and Invasion of Human Colon Cancer Cells

**DOI:** 10.1155/2013/159786

**Published:** 2013-12-10

**Authors:** Jung-Yu Kan, Ya-Ling Hsu, Yen-Hsu Chen, Tun-Chieh Chen, Jaw-Yuan Wang, Po-Lin Kuo

**Affiliations:** ^1^Graduate Institute of Clinical Medicine, College of Medicine, Kaohsiung Medical University, Kaohsiung 807, Taiwan; ^2^Department of Surgery, Division of Gastrointestinal and General Surgery, Kaohsiung Medical University Hospital, Kaohsiung 807, Taiwan; ^3^Graduate Institute of Medicine, College of Medicine, Kaohsiung Medical University, Kaohsiung 807, Taiwan; ^4^Department of Internal Medicine, Division of Infectious Diseases, Kaohsiung Medical University Hospital, Kaohsiung 807, Taiwan; ^5^Faculty of Medicine, College of Medicine, Kaohsiung Medical University, Kaohsiung 807, Taiwan; ^6^Cancer Center, Kaohsiung Medical University Hospital, Kaohsiung 807, Taiwan; ^7^Department of Medical Research, Kaohsiung Medical University Hospital, Kaohsiung 807, Taiwan

## Abstract

Gemifloxacin (GMF) is an orally administered broad-spectrum fluoroquinolone antimicrobial agent used to treat acute bacterial exacerbation of pneumonia and bronchitis. Although fluoroquinolone antibiotics have also been found to have anti-inflammatory and anticancer effects, studies on the effect of GMF on treating colon cancer have been relatively rare. To the best of our knowledge, this is the first report to describe the antimetastasis activities of GMF in colon cancer and the possible mechanisms involved. Results have shown that GMF inhibits the migration and invasion of colon cancer SW620 and LoVo cells and causes epithelial mesenchymal transition (EMT). In addition, GMF suppresses the activation of NF-**κ**B and cell migration and invasion induced by TNF-**α** and inhibits the TAK1/TAB2 interaction, resulting in decreased I**κ**B phosphorylation and NF-**κ**B nuclear translocation in SW620 cells. Furthermore, Snail, a critical transcriptional factor of EMT, was downregulated after GMF treatment. Overexpression of Snail by cDNA transfection significantly decreases the inhibitory effect of GMF on EMT and cell migration and invasion. In conclusion, GMF may be a novel anticancer agent for the treatment of metastasis in colon cancer.

## 1. Introduction

Colon cancer is the leading cause of cancer morbidity and mortality and the third most lethal malignancy worldwide [1, 2]. The incidence rate of colon cancer has increased worldwide over the last 20 years, due to changes in the environment, life styles, and nutritional habits [3, 4]. About 50% of patients with colon cancer will develop metastasis, which is defined as colon cancer cells migrating to and invading organs such as the lungs and liver [5]. Metastasized cancer is particularly lethal and challenging because it is highly resistant to radiation and conventional chemotherapeutic agents, and only one-fourth of patients with metastasis can be treated by surgery [6]. Consequently, novel therapeutic agents are needed to deal with the increasing incidence of the disease, to advance the efficacy of chemotherapy in human colon cancer and increase the number of alternative regimens.

It has been widely reported that persistently activated nuclear factor *κ*B (NF-*κ*B) in tumor cells plays a critical oncogenic role in modulating malignancy transformation and cancer progression [7, 8]. NF-*κ*B signaling, induced by various growth factors, inflammatory factors, and genetic transfection, promotes cancer invasion and metastasis. Inhibition of NF-*κ*B by genetic knockdown or chemical inhibitors has been shown to decrease cancer cell proliferation, migration, invasion, and metastasis and to reduce the chemoresistance of cancer cells to anticancer drugs [9, 10]. Epithelial mesenchymal transition (EMT) is an important process in cancer development, enabling cancer cells to metastasize [11, 12]. Snail, an important transcription factor, has been reported to be involved in the regulation of EMT by repressing the expression of the E-cadherin gene [13]. Recent study has demonstrated that the NF-*κ*B-related cascade modulates Snail expression, leading to EMT in various cell types [14]. Therefore, we hypothesized that inhibition of NF-*κ*B would suppress tumor cell metastasis through inhibition of the downstream target Snail.

Gemifloxacin (GMF) is a fluoroquinolone antimicrobial agent which acts through inhibiting bacterial DNA gyrase and topoisomerase IV [15]. The treatment indications for GMF are community-acquired pneumonia and acute exacerbation of chronic bronchitis [16]. Fluoroquinolones have been reported to decrease tumor growth and DNA synthesis resulting in S- and G2/M-phase cell cycle arrest [17, 18]. This type of antibiotics has also been reported to induce cell apoptosis by causing mitochondrial dysfunction in lung, bone, bladder, and prostate cancer cells [19]. The synergistic effect of GMF on the enhancement of the cytotoxicity of other chemotherapeutic agents, such as doxorubicin and etoposide, has also been reported. In addition, fluoroquinolones have also been reported to reduce the production of inflammatory cytokines/chemokine induced by lipopolysaccharide [20]. In this study, we investigated the anticancer effects of GMF on human colon cancer, with an emphasis on inhibition of cell migration, invasion, and inflammatory stimulation.

## 2. Materials and Methods

### 2.1. Cell Culture and Cell Viability Assay

The human colorectal adenocarcinoma cell line SW620, together with the LoVo cell line, was purchased from the Bioresource Collection and Research Center in Taiwan. SW620 cells were cultured in Leibovitz's L-15 medium (Life Technologies, Inc., Grand Island, NY), supplemented with 10% FBS, 0.1 mg/mL streptomycin, and 100 units/mL penicillin (Life Technologies, Inc., Grand Island, NY), and incubated at 37°C. LoVo cells were cultured at 37°C in a humidified 95% air-5% CO_2_ atmosphere in F12 medium supplemented with 10% FBS. For the cell viability assay, cells (5 × 10^4^/well) were plated in 96-well culture plates. After 24 hours of incubation, the cells were treated with vehicle control (0.1% DMSO) or various concentrations of GMF or TNF-*α* for 48 hours. Viability of the SW620 and LoVo cells was determined by Premixed WST-1 Cell Proliferation Reagent (Clontech Laboratories Inc., Mountain View, CA, USA) according to the manufacturer's instructions.

### 2.2. Scratch Wound-Healing Assay, Cell Migration, and Invasion Assay

The SW620 and LoVo cells were allowed to grow to full confluence in 24-well plates. The following day, a uniform scratch was made down the center of the well using a micropipette tip, followed by washing once with PBS. Various concentrations of GMF were added to the respective wells for the indicated times. Photographic imaging was performed using a Nikon inverted microscope. Quantitative migration and invasion assays were conducted using a QCM 24-well Cell Migration Assay and Invasion System (Millipore Corp., Billerica, MA, USA). Briefly, 1 × 10^5^ SW620 and LoVo cells were seeded into the top chamber and treated with different concentrations of GMF. Ten percent of FBS or TNF-*α* was added to the bottom wells for 48 hours as the chemoattractant. At the end of the treatment, the cells were poststained with CyQuant GR dye in cell lysis buffer for 15 minutes at room temperature. Fluorescence of the migratory and invading cells was then read using a fluorescence plate reader at excitation/emission wavelengths of 485/540 nm.

### 2.3. Immunoblot/Immunoprecipitation

Cells (8 × 10^6^/dish) were seeded in a 10 cm dish. After 24 hours of incubation, the cells were treated with various concentrations of GMF for the indicated times. Total cell extracts were prepared in RIPA lysis buffer (Millipore Corp). Equivalent amounts of protein were resolved by SDS-PAGE and transferred to PVDF membranes. After the membranes had been blocked in Tris-buffer saline containing 0.05% Tween 20 (TBST) and 5% nonfat powdered milk, they were incubated with primary antibodies at 4°C for 1–16 hours. Following three 5-minute washes with TBST, the membranes were incubated with horseradish peroxidase-labeled secondary antibody for 1 hour and then washed again. Detection was performed using an enhanced chemiluminescence blotting detection system (Millipore Corp).

For TAK1 immunoprecipitation, cell lysates (200 *μ*g of total protein) were incubated with 2 *μ*g of anti-TAK1 overnight and then 20 *μ*L of protein A-agarose beads (Millipore Corp., Billerica, MA, USA) for 2 h at 4°C. Association of TAK1 with TAB2 was detected by incubating the blots with anti-TAB2 antibodies (Cell Signaling).

### 2.4. NF-*κ*B DNA Binding Assay

NF-*κ*B activity was measured using an ELISA-based kit according to the manufacturer's specifications (Active Motif, Carlsbad, CA). Briefly, nuclear extracts containing NF-*κ*B were prepared using a Nuclear Extract kit (Active Motif, Carlsbad, CA), and the capture was accomplished by binding to a consensus oligonucleotide (5′-GGGACTTTCC-3′) immobilized on a 96-well plate (Millipore Corp). The p65 subunit of NF-*κ*B was determined in a colorimetric reaction using a specific primary antibody and a secondary horseradish peroxidase-conjugated antibody. Spectrophotometric data were expressed as a ratio of the absorbance of each experimental condition compared with control cells exposed to vehicle alone.

### 2.5. Gene Knockdown and Overexpression

SW620 cells were transfected with either 0.5 *μ*g of the Snail-expressing plasmid or control plasmid by Lipofectamine 2000. Snail cDNA overexpressing SW620 stable colonies were established by G418 selection.

### 2.6. Statistical Analysis

Data were expressed as the mean ± SD of three determinations. Statistical comparisons of the results were made using analysis of variance (ANOVA). Significant differences (*P* < 0.05) between the means of the two test groups were analyzed by Student's *t* test.

## 3. Results

### 3.1. GMF Inhibited Cell Migration and Invasion in SW620 and LoVo Cells

**Figure 1:**
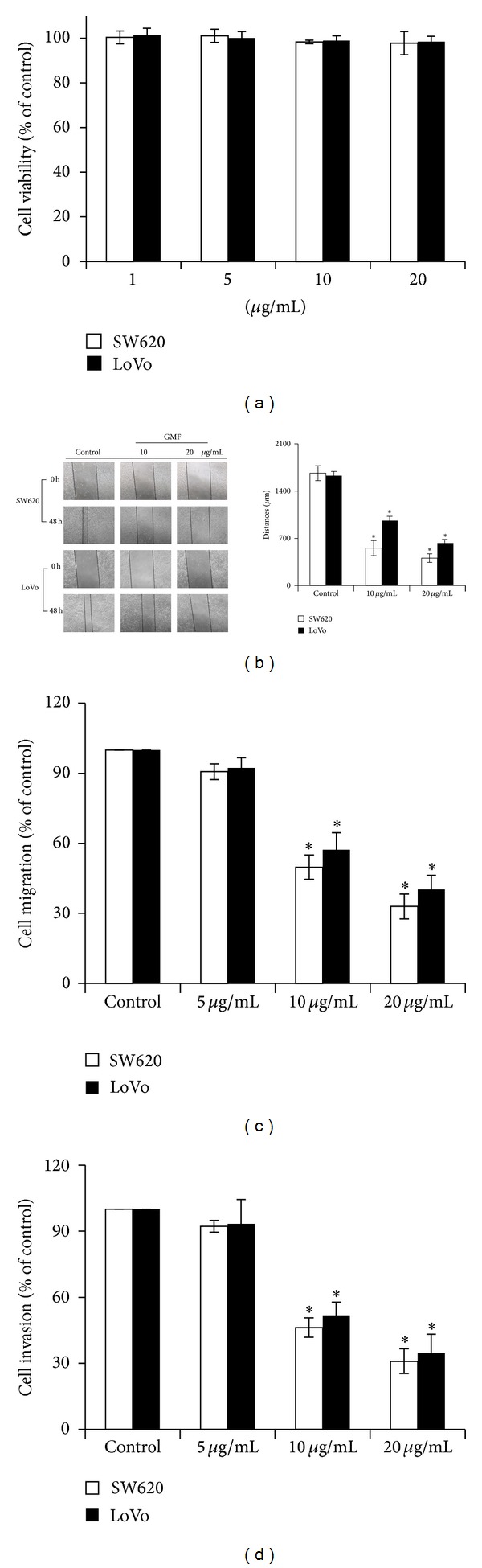
GMF decreased cell migration and invasion in SW620 and LoVo colon cancer cells. (a) The effect of GMF on viability of the SW620 and LoVo cells. GMF reduced cell migration as determined by wound healing analysis (b) and the transwell system (c). (d) GMF decreased cell invasion. The migration and invasion abilities of the SW620 and LoVo cells were quantified by QCM 24-well Cell Migration and Invasion assay kits, as described in Section 2. Ten percent of FBS acted as the chemoattractant for cancer migration and invasion. All results are representative of at least three independent experiments. Each value is the mean ± SD of three determinations. The asterisk (∗) indicates a significant difference between the two test groups, as analyzed by Student's *t* test (*P* < 0.05).

We first assessed the effects of GMF on the viability of SW620 and LoVo cells. As shown in Figure 1(a), GMF did not affect the viability of SW620 and LoVo cells at concentrations ranging from 1 to 20 *μ*g/mL. However, GMF exhibited an inhibitory effect on the migration of SW620 and LoVo cells, as determined by wound-healing assay (Figure 1(b)). In addition, quantitative transwell analysis also revealed that GMF decreased the migration of SW620 and LoVo cells in a dose-dependent manner (Figure 1(c)). Next, we assessed the effect of GMF on the invasive ability of colon cancer cells. Compared to vehicle-treated cells, GMF treatment attenuated SW620 and LoVo cell invasion in a dose-dependent manner after treatment for 48 hours (Figure 1(d)).

### 3.2. GMF Decreased Snail Expression and Caused EMT in SW620 Cells

**Figure 2:**
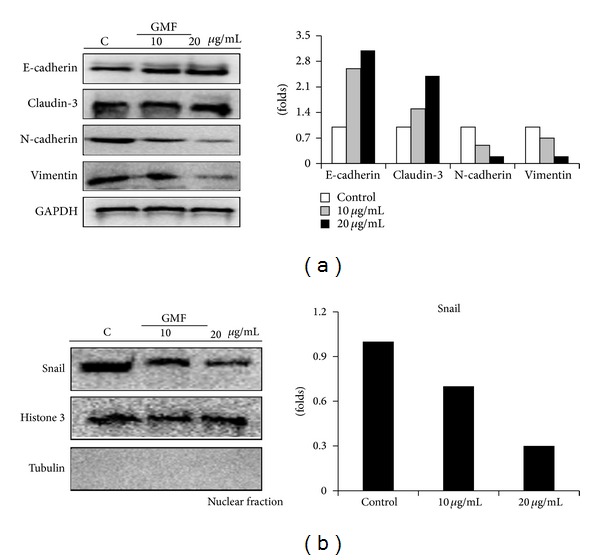
GMF changed the expressions of mesenchymal markers in SW620 cells. (a) GMF increased the levels of epithelial protein and reduced mesenchymal factors in the SW620 cells. (b) GMF reduced Snail levels. Cells were treated with various concentrations of GMF for 24 hours, and the protein expression was assessed by immunoblot assay. All results are representative of at least three independent experiments.

EMT is a critical process in the development of invasive cancer cells, and restoration of the epithelial-like characteristics can reduce tumor invasive capacity [21]. We assessed the effect of GMF on EMT markers in SW620 cells and found that GMF treatment enhanced epithelial markers, including E-cadherin and claudin-3 levels. In contrast, GMF reduced mesenchymal markers such as vimentin and N-cadherin expression (Figure 2(a)).

Next, we investigated whether Snail, an important transcription factor in regulating cell migration and EMT, was involved in the GMF-mediated switch of EMT phenotype. Immunoblot analysis revealed that protein levels of Snail in the nuclei were reduced by GMF treatment of the SW620 cells (Figure 2(b)).

### 3.3. The Role of Snail on GMF-Mediated Inhibition of Cell Migration and EMT

**Figure 3:**
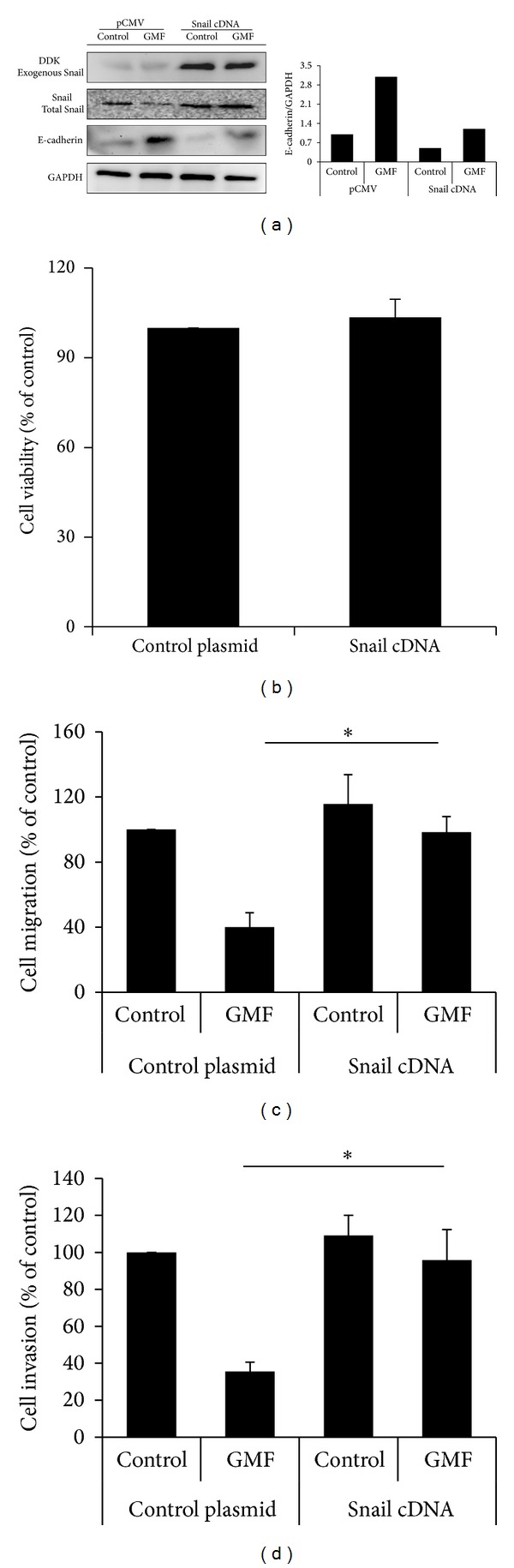
The role of Snail in GMF-mediated cell migration and invasion inhibition. (a) The effect of overexpression of Snail in GMF-treated SW620 cells. (b) Snail overexpression did not affect viability of SW620 cells. Snail overexpression decreased the inhibitory effect of GMF on cell migration (c) and invasion (d). Cells were transfected with pCMV or pSnail plasmids, and stable colonies were established by G418 selection. Snail-overexpressing cells were treated with GMF (20 *μ*g/mL) (24 hours for E-cadherin), and various protein levels were assessed by immunoblot assays. Each value is the mean ± SD of three determinations, and all results are representative of at least three independent experiments. The asterisk (∗) indicates a significant difference between the two test groups, as analyzed by Student's *t* test (*P* < 0.05).

To investigate the role of Snail on GMF-mediated cancer migration inhibition and EMT, we generated overexpressing Snail human colon cancer SW620 cells steadily expressing Snail cDNA and then confirmed the exogenous protein expression by immunoblot (Figure 3(a)). Snail overexpression was not found to affect the viability of SW620 cells after 48 h analysis (Figure 3(b)). We then examined the effect of GMF on the Snail-overexpressing SW620 cells. As shown in Figures 3(c) and 3(d), Snail overexpression blocked the effects of GMF on cell migration and invasion. In addition, the effects of GMF on the upregulation of E-cadherin were also blocked by Snail overexpression (Figure 3(a)). These data suggest that GMF decreases cancer cell progression by inhibiting Snail.

### 3.4. GMF Inhibits Constitutive and Inducible NF-*κ*B Nuclear Translocation and Activity in SW620 Cells

**Figure 4:**
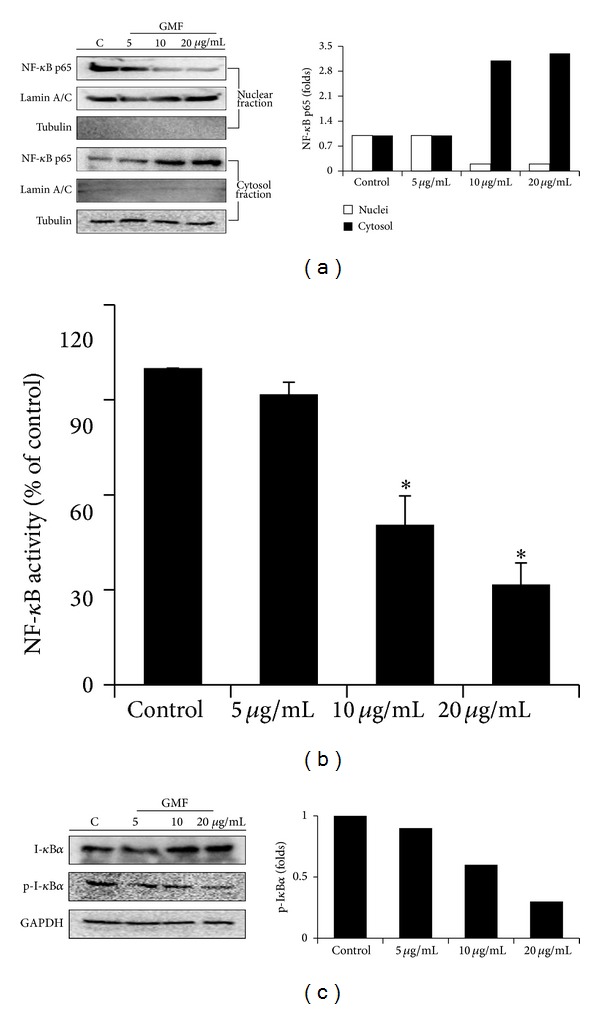
GMF inhibited NF-*κ*B activity in SW620 cells. GMF decreased NF-*κ*B nuclear translocation (a) and activity (b) in the SW620 cells. (c) GMF increased I-*κ*B*α* levels in the cytosol. Cells were treated with various concentrations of GMF. Nuclear and cytoplasm fractions were separated by a nuclear extract kit, and protein expressions were assessed by immunoblot assay. The DNA binding activity of NF-*κ*B in the nuclear fraction was assessed by a Trans-AM ELISA kit. All results are representative of at least three independent experiments. Each value is the mean ± SD of three determinations. The asterisk (∗) indicates a significant difference between the two test groups, as analyzed by Student's *t* test (*P* < 0.05).

Increased NF-*κ*B signaling has been reported to act as a critical regulator and transcriptional activator in colon cancer [22]. We consequently tested whether GMF has a direct impact on NF-*κ*B activation. As shown in Figure 4(a), the protein level of NF-*κ*B in the nuclei was decreased by GMF treatment (Figure 4(a)). In addition, NF-*κ*B activity (nuclear fraction) in SW620 cells decreased after GMF treatment (Figure 4(b)). Because phosphorylation and degradation of I*κ*B by I*κ*B kinase (IKK) is an important step in the process of NF-*κ*B activation, we investigated the effect of GMF on the status of I*κ*B. GMF decreases the phosphorylation of I*κ*B and increases the amount of I*κ*B in the SW620 cells, suggesting that the effect of GMF on NF-*κ*B operates by decreasing I*κ*B phosphorylation (Figure 4(c)).

**Figure 5:**
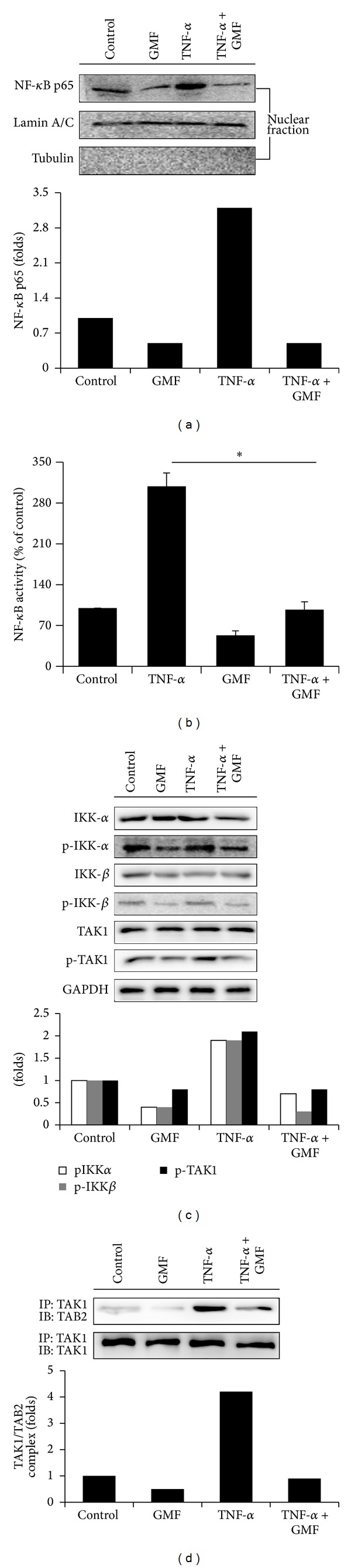
GMF inhibited inducible NF-*κ*B activity in SW620 cells. GMF reduced TNF-*α*-mediated NF-*κ*B nuclear translocation (a), activation (b), IKK phosphorylation (c), and TAK1/TAB2 interaction (d). Cells were pretreated with GMF (20 *μ*g/mL) for 1 hour; then TNF-*α* (20 ng/mL) was added for another 3 hours. Protein expression was assessed by immunoblot, and protein-protein interaction was examined by immunoprecipitation. The DNA binding activity of NF-*κ*B in the nuclear fraction was assessed by a Trans-AM ELISA kit. All results are representative of at least three independent experiments. Each value is the mean ± SD of three determinations. The asterisk (∗) indicates a significant difference between the two test groups, as analyzed by Student's *t* test (*P* < 0.05).

Inflammatory factors such as TNF-*α* are thought to be major causes of elevated NF-*κ*B in cancer cells [23]. We therefore investigated whether GMF also inhibited TNF-*α*-mediated NF-*κ*B activation. As shown in Figure 5(a), TNF-*α* (20 ng/mL) increases the nuclear translocation of NF-*κ*B in SW620 cells. In addition, GMF also reduces the DNA binding activity of NF-*κ*B in a dose-dependent manner after 3 hours of treatment (Figure 5(b)). These data suggest that GMF is a potential inhibitor of oncogenic transcription factors.

Because IKK-*α*/*β* are upstream activator of I*κ*B in the NF-*κ*B signal pathway, we assessed the effect of GMF on TNF-*α* treated colon cancer cells. It was found that GMF markedly decreases TNF-*α*-induced IKK-*α*/*β* phosphorylation, without affecting the total amounts of IKK-*α*/*β* (Figure 5(c)). Furthermore, because TAK1 has been implicated in the regulation of IKK-*α*/*β* phosphorylation by inflammatory cytokines, we further examined the effect of GMF on the TNF-*α* induced phosphorylation of TAK1. Our results show that TNF-*α* treatment increases TAK1 phosphorylation, which was significantly inhibited by GMF. To determine whether GMF decreases TAK1 phosphorylation by decreasing the interaction of the TAK1 with TAB2, we assessed the association of TAK1/TAB2 by immunoprecipitation. Binding of TAK1 to TAB2 was observed after TNF-*α* treatment, but the interaction was significantly decreased after treatment with GMF (Figure 5(d)). These findings suggest that GMF suppresses TNF-*α*-induced NF-*κ*B activation by down-regulation of the TAB2/TAK1-mediated NF-*κ*B pathway.

### 3.5. GMF Decreases Inflammatory TNF-*α*-Mediated Cell Migration, Invasion, and EMT in SW620 Cells

**Figure 6:**
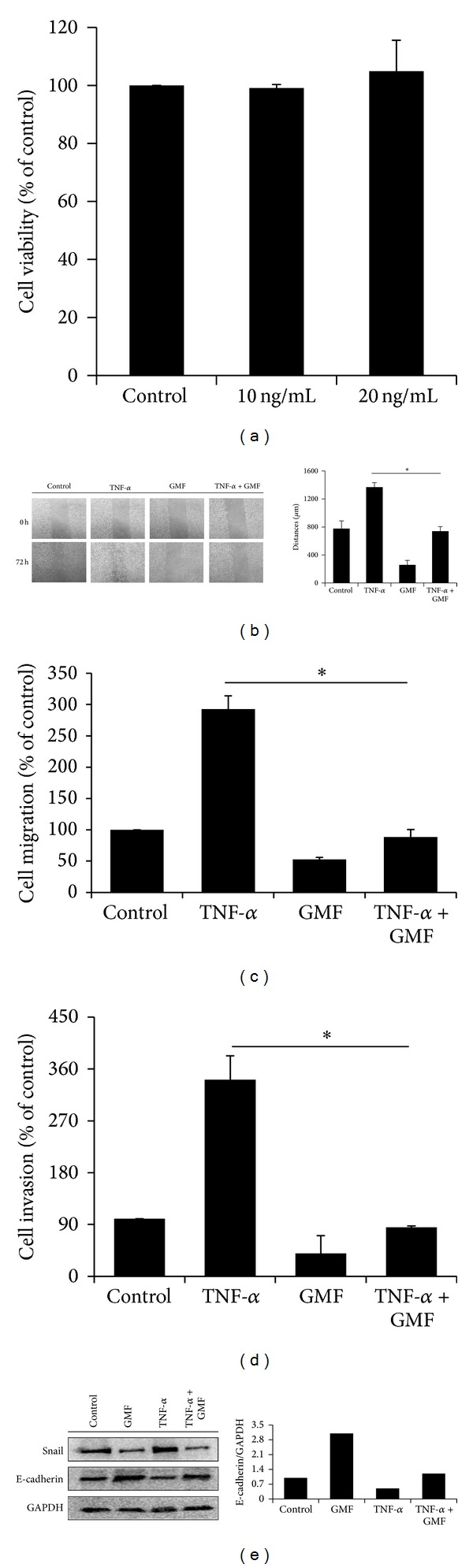
GMF reduced inflammatory factor TNF-*α*-mediated cell migration, invasion, and EMT in SW620 cells. (a) The effect of TNF-*α* on cell viability. GMF inhibited cell migration, as determined by wound-healing (b) and transwell system (c) and invasion (d) and EMT (e) were induced by TNF-*α*. The migration and invasive abilities of the SW620 cells were quantified by QCM 24-well Cell Migration and Invasion assay kits. TNF-*α* (20 ng/mL) acted as a chemoattractant of cancer migration and invasion. Each value is the mean ± SD of three determinations. The asterisk (∗) indicates a significant difference between the two test groups, as analyzed by Student's *t* test (*P* < 0.05).

The inflammatory factor TNF-*α* is known to promote metastasis by enhancing EMT and invasiveness in colon cancer [23]. We therefore investigated whether GMF decreases the enhancement of TNF-*α* on the biologic events of cancer progression. Since TNF-*α* did not affect the viability of SW620 cells (Figure 6(a)), we further assessed the effect of GMF on cell migration and invasion. As shown in Figures 6(b) and 6(c), treatment of the SW620 cells with TNF-*α* (20 ng/mL) increases cell migration, but this effect is abrogated by GMF. In addition, GMF also abolishes TNF-*α*-induced cell invasion (Figure 6(d)). Furthermore, treatment with GMF also inhibits Snail upregulation and E-cadherin downregulation induced by TNF-*α* in SW620 cells (Figure 6(e)).

## 4. Discussion

Discovering new uses for preexisting drugs with well-defined profiles, such as side effects, pharmacokinetics, and pharmacodynamics, may prove to be beneficial for patients. These drugs have the potential and offer the advantage of extensive clinical experience in other therapeutic areas [24]. Therefore, their potential value in the prevention of metastasis is high. The current study shows that GMF, a clinical used antibiotic, decreases the metastasis of colon cancer SW620 and LoVo cells by decreasing cell migration and invasion. This study also investigates the effect of GMF in reversing inflammatory cytokine-mediated cancer progression. Our findings suggest that GMF is capable of preventing the progression of colon cancer.

The NF-*κ*B signaling pathway is involved in the pathogenesis of various cancers, including colon cancer [25, 26]. Elevated activation of NF-*κ*B and upstream triggers such as TNF-*α* supports the hypothesis that this pathway plays a crucial role in cancer development [27, 28]. When inactive, NF-*κ*B dimers are associated with inhibitory I*κ*B proteins and sequestered in the cytoplasm. Stimulus-induced phosphorylation and ubiquitination of I*κ*B by the I*κ*B kinase complex have been reported to result in proteasome-mediated degradation, which in turn causes nuclear translocation and DNA binding of NF-*κ*B [29, 30]. The phosphorylation of I*κ*B is catalyzed by I*κ*B*α* kinase (IKK), which is necessary for NF-*κ*B activation [30]. TAK1 has also been indicated to be an upstream activator of IKK in the canonical NF-*κ*B signaling pathway, activated by inflammatory cytokines through its interaction with TAB1 and TAB2 [31, 32]. The current study reveals that GMF not only decreases the constitutive NF-*κ*B nuclear translocation and activity but also blocks inducible NF-*κ*B activation mediated by TNF-*α*. The inhibitory effect of GMF on inflammatory factor NF-*κ*B-mediated cancer progression was also revealed by decreasing the oncogenic potential of TNF-*α*-mediated cell migration, invasion, and EMT after GMF treatment of the SW620 cells. This inhibitory effect of GMF on NF-*κ*B is associated with a decrease in the interaction of TAK1 and TAB2, which in turn reduces IKK activation, thereby resulting in I*κ*B phosphorylation and degradation. These data suggest that GMF is a potential inhibitor targeting both constitutive and inducible activation of NF-*κ*B.

Snail, a transcription factor, is a critical modulatory factor of cell motility which operates by changing the cell phenotype from an epithelial characteristic to mesenchymal. Its expression has been reported to be elevated in several cancer types, including colon cancer [33, 34]. NF-*κ*B signaling is known to be a regulator of Snail in various types of cells [14]. Triggering of the NF-*κ*B pathway regulates EMT in human cancers by increasing Snail [14, 35]. In addition, Snail inhibits the expression of tight junction E-cadherin protein by binding to E2-box type elements within its promoter, resulting in EMT [13, 36]. E-cadherin downregulation and EMT have been implicated in the increase of metastatic ability and are strongly associated with a poor prognosis [37]. In contrast, mesenchymal-like tumors can revert to an epithelial-like characteristic via the MET process, resulting in a reduction in metastatic capacity [21, 38]. We found that GMF had a significant inhibitory effect on Snail expression, which was consistent with the blockade of NF-*κ*B by GMF. The inhibition of Snail directly contributes to the restoration of E-cadherin and inhibition of mesenchymal gene markers (N-cadherin and vimentin). The ectopic expression of Snail blocks antimigration, and E-cadherin upregulates the effect of GMF in SW620 cells. These results suggest that inhibition of Snail may be pivotal for GMF-mediated E-cadherin induction and promotion of EMT.

Taken together, our findings provide strong evidence that GMF decreases colon cancer metastasis. The molecular mechanism of GMF in reducing the capacity of cell migration and invasion by decreasing NF-*κ*B contributes to the change of metastatic phenotypes in colon cancer. Therefore, GMF may serve as a potential agent for the development of therapies against early metastatic events in colon cancer.
